# Comparison of Nutritional Knowledge, Attitudes and Practices between Urban and Rural Secondary School Students: A Cross-Sectional Study in Sabah, East Malaysia

**DOI:** 10.3390/foods10092037

**Published:** 2021-08-30

**Authors:** Mohammad Halim Bin Jeinie, Rhanye Mac Guad, Marion M. Hetherington, Siew Hua Gan, Yin Nwe Aung, Wu Yuan Seng, Constance Liew Sat Lin, Ramlah George, Waidah Sawatan, Norazmir Md Nor, Nang Kham Oo Leik, Mohd Nazri Bin Mohd Daud, Shutie Fazila Guad

**Affiliations:** 1 Faculty of Food Science and Nutrition, Universiti Malaysia Sabah, Kota Kinabalu 88400, Sabah, Malaysia; ramlah@ums.edu.my; 2 Department of Biomedical Science and Therapeutics, Faculty of Medicine & Health Science, Universiti Malaysia Sabah, Kota Kinabalu 88400, Sabah, Malaysia; 3 School of Psychology, University of Leeds, Leeds LS2 9JT, UK; m.hetherington@leeds.ac.uk; 4 School of Pharmacy, Monash University Malaysia, Bandar Sunway 47500, Selangor Darul Ehsan, Malaysia; Gan.SiewHua@monash.edu; 5 Faculty of Medicine & Health Sciences, UCSI University, Jalan Menara Gading, UCSI Heights, Cheras 56000, Kuala Lumpur, Malaysia; yinna@ucsiuniversity.edu.my; 6 Department of Biochemistry, School of Medicine, Faculty of Medicine, Bioscience and Nursing, MAHSA University, Jenjarom 42610, Selangor, Malaysia; sengwu_21@yahoo.com; 7 Medical Based Department, Faculty of Medicine & Health Science, Universiti Malaysia Sabah, Kota Kinabalu 88400, Sabah, Malaysia; constance.liew@ums.edu.my; 8 Department of Nursing, Faculty of Medicine & Health Science, Universiti Malaysia Sabah, Kota Kinabalu 88400, Sabah, Malaysia; waidah@ums.edu.my; 9 Integrative Pharmacogenomics Institute (iPROMISE), Universiti Teknologi MARA (UiTM), Puncak Alam 42300, Selangor, Malaysia; azmir2790@uitm.edu.my; 10 Department of Reproductive Health, Faculty of Medicine & Health Science, Universiti Malaysia Sabah, Kota Kinabalu 88400, Sabah, Malaysia; nangkhamooleik@ums.edu.my; 11 Department of Community and Family Medicine, Faculty of Medicine and Health Science, Universiti Malaysia Sabah, Kota Kinabalu 88400, Sabah, Malaysia; mohd.nazri@ums.edu.my; 12 Sabah State Education Department, Kota Kinabalu 88450, Sabah, Malaysia; fazila_shutie@yahoo.com

**Keywords:** knowledge, attitude, practice, nutrition, urban, rural, Sabah students

## Abstract

Nutritional knowledge, attitudes and practice (KAP) may guide healthy meal choices. Here, nutritional KAP was compared across school students in Sabah based on locality and gender. A cross-sectional survey of students aged 15–19 years was conducted using multistage sampling. Nutritional KAP was measured via questionnaire. Anthropometric measures of weight and height were taken in person to calculate body mass index (BMI). Among the 994 participants, 80% were urban and 60% were female (mean age 16.5 ± 0.6 yr). Most were of *Kadazan-Dusun* (23%) ethnicity. Measured height for age Z score (HAZ) and BMI for age Z score (BAZ) differed between urban and rural students (−1.2 ± 0.8 versus −1.5 ± 0.7 for HAZ; *p* < 0.001; 0.2 ± 1.4 versus −0.1 ± 1.3; *p* = 0.02, respectively). No difference in nutritional knowledge was found, although urban students prioritized having a healthy/balanced diet (59.55% versus 48.50%, *p* = 0.03) and ate daily breakfast (57.4% versus 10.2%, *p* < 0.001) compared to rural. Females scored higher on nutritional knowledge than males (18.9 ± 2.8 vs. 18.1 ± 3.4, respectively, *p* = 0.0001), yet males selected more healthy/balanced foods (63.3% versus 53.3%, *p* = 0.041). The gap remains between nutritional KAP and translating this to healthy eating among adolescents, related to locality and gender.

## 1. Introduction

The Malaysian Education Blueprint 2013–2025 aims to provide an opportunity for eligible citizens to have access to education and standards (quality), reduce the achievement gap (equity) and improve the education system’s effectiveness among individuals of all school levels [1]. Primary education in Malaysia begins at 7 years old, up to 12 years old. Subsequently, students spend a further five years in their secondary education (Forms 1–3: Lower Secondary; Forms 4–6: Upper Secondary). Although the curricula remain standard across Malaysia under the Secondary School Standard Curriculum or *Kurikulum Standard Sekolah Menengah (KSSM)*, there remain some differences in the circumstances in which children receive their education. For example, the context of learning may vary to a certain extent between urban and rural settings.

The Malaysian National Statistical Department define rural schools as those located inside (urban) or outside (rural) a metropolitan area with a population of more than 2500 people [2,3]. Challenges with nutrition (both under- or overnutrition) are common in many countries in the world including Malaysia [4]. Several studies have provided evidence of the challenges among school students in Malaysia. One such study, the National Health and Morbidity Survey (NHMS) reported that among Malaysians aged 10–17 years, the prevalence of obesity had risen from 5.7% in 2011 to 11.9% in 2015 [5]. Poh BK et al. [6] indicated that the data from Nutrition Survey of Malaysian Children (SEANUTS) Malaysia are mostly skewed towards school-aged children from urban areas. In contrast, Khor et al. [7] reported the persistence of underweight and stunting among children in rural areas of Sabah [8] indicating the need for further investigation on the display of the nutritional issues seen in the two geographical regions.

The nutritional issues in Malaysia have been recognized and acknowledged by various government and non-government agencies. For example, the National Plan of Action for Nutrition of Malaysia [NPANM] III (2016–2025) promotes nutritional education in schools. At approximately the same time, the School Meal Programme was implemented by at least three different schools in every state by 2020 and at least six schools in every state by 2025 [9] indicating the importance of the issue.

Clearly, there are multiple, complex factors which influence the nutritional status of individuals, including family income [10], parental education level [11] and specifically nutritional education [12]. Scaglioni et al. (2018) [13] proposed that understanding of healthy eating and establishing sustainable dietary patterns can and should be cultivated at a very young age where dietary habits established in childhood tend to track into later life. Individuals are also affected by culture, as evidenced by differences in obesity prevalence observed across nations, cultures and ethnicities. For instance, an analysis of 2011–2016 USA National Health and Nutrition Examination Surveys indicated that Filipino (adjusted odd ratio or AOR 2.79; 95% confidence interval or CI 1.30–6.00), Japanese/Korean (AOR 2.55; 95% CI 1.21–5.38), Southeast Asian (AOR 2.54; 95% CI 1.63–3.94) and South Asian (AOR 2.10; 95% CI 1.01–4.36) children aged between 6 and 19 years old were more likely to have overweight/obesity than those from the similar age group in China [14]. Similarly, dietary patterns vary widely by race and ethnicity even within a single country, with for instance, varying frequency of vegetable intake based on ethnic group [15].

Sabah is one of the 14 states of Malaysia located on the Borneo Island, East Malaysia. A multi-ethnic population resides there, with the *Kadazan-Dusun* and *Bajau* being the predominant ethnic clusters which in turn consist of more than 100 sub-ethnicities [16]. Given the powerful link between ethnicity, culture and food, it is plausible that there are substantial differences between communities in terms of attitudes, beliefs and practices which influence nutritional knowledge and dietary patterns in the daily lives of the populace.

Given that the nutritional status of secondary school students in urban areas has been investigated [3], there is a gap in knowledge of nutrition education for individuals residing in rural areas. Of the few studies which have been conducted in this context, it has been reported that school-aged children in East Malaysia have both poor nutritional knowledge and low socioeconomic status [3]. There are clear detrimental effects of having a poor nutrition on the physical growth and development between deprived and affluent children where nutritionally deficient diets tend to affect educational performance [17]. Further investigations are needed within rural areas to understand the interplay between knowledge of nutrition, attitudes and practices (KAP) among secondary school students between urban and rural areas in Sabah. A better understanding of the inter-relationship will benefit future development and implementation of strategic and proactive policies to promote healthy eating through implementation of better nutritional knowledge and the provision of healthy meals to secondary school students. We hypothesized that KAP would differ by urban and rural contexts, and by gender. It was predicted that the greater interest girls have in nutrition, healthy eating and remaining lean would produce higher KAP scores and healthier eating in girls than boys. This is based on previous evidence that girls, more than boys, are interested in attaining a healthy body shape and in using dietary knowledge to pursue healthy eating [18].

## 2. Materials and Methods

### 2.1. Ethics Approval

Ethical approval was obtained from the Education Department of Sabah (JPNSBH.SPS.100-2/1/1Jld.8) and Universiti Malaysia Sabah ethics committee (UMS/FPSK6.9/100-6/1/95) which complies with the Declaration of Helsinki. Written informed consents were obtained from students and their parents/guardians prior to participation. The participants could withdraw from the study at any time.

### 2.2. Study Design

**Figure 1:**
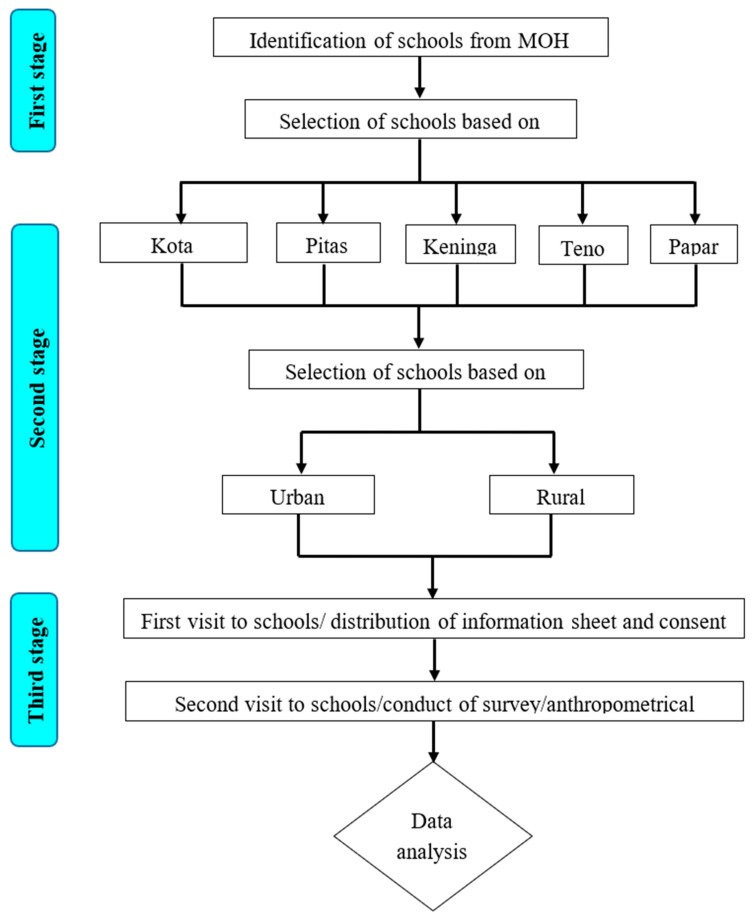
Multistage cluster sampling process for student recruitment survey questionnaire.

This is a cross-sectional survey conducted between May and August 2019. It is based on a two-stage sampling process. A list of all government secondary schools in Sabah and Sarawak was compiled from the latest Malaysia Ministry of Education (MOE) database. The schools were first classified by district (for example Sabah: Kota Kinabalu and Pitas) and then by location (urban, rural) (refer diagrammatic representation on Figure 1). The step is done to ensure that the sampling process took into account the distribution of schools and students across urban, rural, other remote areas and various districts of the student population in each state. This move will ensure enrolling a good spread of rural and urban schools with a sufficiently large recruitment, in order to have a broader representation of the student population. The school groupings were then placed into a comprehensive ordered list for sampling. The probability of a school being selected was calculated based on the size of its enrolment in relation to the total enrolment for the state and the number of schools needed for the study. Therefore, schools with large number of student populations are more likely to be selected because their students represent a larger proportion of the student population to be sampled from.

### 2.3. Study Participants and Sample Size Determination

Sample size estimation was calculated using the Cochran (1963) formula to achieve an 80% power with a 5% margin of error and 95% confidence level [19]. Given that norms and averages for KAP in this study population are unknown, the estimated sample size was thus calculated by assuming that 50% of the population have a baseline knowledge about nutrition to yield the largest sample size possible. Based on the calculation, a minimum number of 384 students was required for the study. However, an additional 20% was added to the number in case of dropouts. Respondents were included if they matched the required criteria such as being a Malaysian citizen, aged from 16 to 20 and studying in Forms 4 (17 years old), 5 (18 years old) or 6 (19 years old). After a thorough screening, a total of 994 Forms 4–6 secondary school students from both urban and rural areas of Sabah were included in the survey and their responses recorded.

### 2.4. Data Collection Process

Prior to study commencement, the selected schools were contacted, and the proposed study dates given for distribution of questionnaires. During data collection, all students were gathered in a classroom or hall and were briefed about the purpose of the study. Besides getting their written consents, they were also required to have consents from their parents/guardians.

The study employed a paper and pencil self-administered questionnaire with the students seated far apart from one another to avoid any potential bias. An assistant was available to ensure the questionnaires were completed and all questions were answered. The time taken to complete the questionnaire was approximately 30 min/participant on average.

Anthropometric measurements were taken for weight, height and body mass index (BMI) conducted after completion of questionnaires (refer diagrammatic representation on Figure 1). The body mass index (BMI) was calculated as weight (in kg) divided by height (in metres squared) to yield a number in kg/m^2^. The BMI for adults was utilized and adapted with two cut-off point for Asian as suggested by the World Health Organization (WHO) (2000). The participants were categorized as having underweight (BMI < 18.5 kg/m^2^), normal weight (BMI = 18.5–22.9 kg/m^2^), overweight (BMI = 23.0–24.9 kg/m^2^) and obese (BMI ≥ 25.0 kg/m^2^) status [20]. The anthropometric data of height for age z score (HAZ) and BMI for age Z score (BAZ) was re-analyzed using WHO AnthroPlus software based on WHO 2007 Growth reference for children and adolescents between 5 and 19 years old; HAZ values less than 2SD were considered stunting. Overweight and obesity were classified as BAZ values greater than 1SD and 2SD, respectively, while thinness was defined as BAZ values less than 2SD [20].

### 2.5. Questionnaire Design

The questionnaire was adapted from a previous study in Malaysia [21], and a final structured questionnaire was utilized as a tool for data collection following a pilot study on 30 local secondary school students. It consisted of 31 questions that were divided into four sections comprising (A) sociodemographics (eight items) (B) nutritional knowledge (25 items), multiple choice (five options) covering physiological function of food, the relationship between diet/nutrient and disease, national guidance (food pyramid) on daily serving requirements (C) attitudes and behaviors (six items) scored on a 5-point Likert-type format, from ‘strongly disagree’, to ‘strongly agree’ and dietary practices and preferences (six items) using a 5 point frequency scale from “every day” to “always” and from “dislike strongly” to “like strongly”.

The total scores were calculated with each correct answer assigned a score of 1 while incorrect responses were given 0 for nutritional knowledge. The scores were classified according to a. <40% (low), b. 41–69% (moderate) and c. ≥70% (high). The mean values (±SD), median and the 25th, 50th and 75th percentiles were computed for both locality of schools and gender. For attitudes, behaviors, practices and preference, a score of 5 for each question reflected a healthier answer while a score of 1 indicated a less healthy answer.

### 2.6. Statistical Analysis

The data were entered into SPSS statistical software program (SPSS version 22, Armonk, NY, USA). The sociodemographic characteristics were presented descriptively as n (%), mean ± SD and median while bivariate analysis was performed to compare scores on questionnaire variables using chi-square tests. Independent sample *t*-tests were used for comparison of normally distributed continuous data, while Mann–Whitney tests were used for not normally distributed continuous data. Univariate analyses were performed to determine the association of KAP with the school locality and gender. Correlations between KAP subscales were analyzed using Spearman correlations. A *p*-value of less than 0.05 was considered as statistically significant.

## 3. Results

### 3.1. Sociodemographics of the Urban and Rural Secondary School Students

Table 1 presents the demographic status of the students. A total of 994 responses (98% response rate) were recorded where 80% students were from urban schools while 20% were from rural schools. Most of the participants were drawn from Form 4 (54.5%) and Form 5 (44.0%) with very few from Forms 3 (0.4%) and 6 (1.5%). The sample was mostly female (59.7%) with a mean age 16.5 ± 0.59 years old. Most students were *Kadazan-Dusun* (23.1%) and *Bajau* (16.7%), reflecting the local population where they are the largest indigenous groups in Sabah. However, a large diversity of the ethnic groups was seen where the rural schools tended to have a higher proportion of students drawn from the *Kadazan-Dusun* group whereas urban schools tended to have a higher proportion of students from the *Bajau* communities. As expected, there was a significant difference in the measured height for age Z score (HAZ) (−1.11 ± 0.82 versus −1.51 ± 1.76 for HAZ; *p* < 0.001) between urban and rural students. In addition, the mean heights for boys and girls were also significantly different (−1.04 ± 0.80 versus −1.51 ± 1.76; *p* = 0.010 and −1.17 ± 0.84 versus −1.64 ± 2.14; *p* < 0.001, respectively). There was a significant difference between urban and rural students in terms of BMI for age Z score (BMIz) score (1.08 ± 0.20 versus 1.12 ± 0.19; *p* = 0.019). Although the BMIz for girls was significantly different (1.1 ± 0.17 versus 1.14 ± 0.15; *p* = 0.024, the difference was not observed among boy students in urban and rural schools.

### 3.2. Nutritional Knowledge of Students Differs with Locality of School and Gender

The nutritional knowledge score of urban students varied from 1 to 24, while that for rural students varied from 11 to 23 (Table 2). Overall, the mean score on nutritional knowledge was similar between urban and rural students (18.59 ± 3.14 and 18.59 ± 2.66 respectively, *p* = 0.986). At 25th percentile, both urban and rural scores on knowledge were similar (a score of 17). Nutritional knowledge score for males varied from 1 to 24, while for females it varied from 7 to 24. In terms of mean score on nutritional knowledge, female students showed significantly higher knowledge as compared to their counterparts (18.9 ± 2.8 vs. 18.1 ± 3.4, respectively, *p* ≤ 0.0001). Both genders scored similarly at 25th and 75th percentiles (19 and 21, respectively).

### 3.3. Nutritional Attitude of Students Differs with Locality of School and Gender

The association between attitudes, locality of schools and gender of secondary school students is outlined in Table 3. Urban students prioritized having a healthy and balanced diet when choosing food compared to rural students (59.55% versus 48.50%, *p* = 0.03). However, fewer rural students reported the need to have a snack in between meals compared to urban students (30.69% versus 38.60%, *p* = 0.008). Additionally, a higher number of rural students do not eat outside their three regular mealtimes of the day (59.41% versus 51.34%, *p* = 0.049). Interestingly, more males choose healthy and balanced foods than their female counterparts (63.29% versus 53.30%, *p* = 0.041).

### 3.4. Nutritional Practices Differ with Locality of School and Gender

Table 4 shows the nutritional practices and their association with locality of schools as well as gender. A higher number of urban students consumed breakfast daily as compared to rural students (72.14% versus 50.25%, *p* < 0.001). Although rural students are associated with higher consumption of vegetables (96.98% versus 90.21%; *p* = 0.024), they drank lesser amounts of milk at least once a week (67.89% versus 66.00%; *p* = 0.002) as compared to urban students. Male students reported to have higher intake of fast foods (71.10% versus 70.09%; *p* = 0.004), less consumption of vegetables (89.60% versus 92.89%; *p* = 0.011) and milk (72.26% versus 73.01%; *p* = 0.005) in at least once a week as compared with female students.

### 3.5. Correlation between KAP Domains

A positive correlation was established between (1) nutritional knowledge and practice (r = 0.18, *p* < 0.001) and (2) nutritional attitude and practice (r = 0.16, *p* < 0.001) among urban students. However, the correlation was not observed among rural students. Additionally, there was no correlation between knowledge and attitude (r = 0.02, *p* = 0.529) among urban and rural students. Nevertheless, a mild but significant association was demonstrated between attitude with practice (r = 0.154, *p* < 0.001) and knowledge with practice (r = 0.160, *p* < 0.001).

In terms of gender, a significant correlation was established between male nutritional knowledge and practice (r = 0.13, *p* = 0.0115) as well as nutritional attitude and practice (r = 0.17, *p* < 0.001). Similarly, the trend was observed in their female counterparts for (1) knowledge and practice (r = 0.19, *p* < 0.001) and for (2) attitude and practice (r = 0.14, *p* < 0.001, Table 5).

## 4. Discussion

To the best of our knowledge, this is the first study conducted in East Malaysia comparing KAP on nutrition between rural and urban secondary school children. As would be expected, there were significant differences in anthropometrics between urban and rural settings with higher HAZ, BMI and BMI for age scores among urban compared to rural students. Despite having high nutritional knowledge in this study, there was only a moderate nutritional attitude and practice level. Furthermore, a modest correlation between knowledge and practice was observed in this study.

Urban students appeared to prioritize having a healthy and balanced diet when choosing food as compared to rural students, more urban students reported the need to have a snack in between meals and regardless of the time of day. However, this may simply reflect differences in opportunity between urban and rural settings, since students with more resource and access to food may prioritize eating for health over eating for fuel. Interestingly, although urban students drank more milk at least once a week compared to rural students, they had less consumption of vegetables. Small, significant differences in nutrition knowledge were found between females and males. However, this small difference in knowledge did not translate into action, since males reported choosing more healthy and balanced foods compared to their female counterparts.

In support of a previous study of nutritional knowledge in Malaysian adolescents, knowledge is relatively high, but nutritional deficiencies, such as anemia also remain high [12]. In the current study, nutrition knowledge did not vary by setting. However, it was expected that nutrition knowledge would be higher in urban students as was reported in Tyrol, Western Austria [22]. A meta-analysis conducted on 74,168 pooled participants aged 2–19 in the United States found that rural children had 26% greater odds of having obesity compared to urban children (odds ratio = 1.26; 95% confidence interval, 1.21–1.32) [23]. In contrast, rural students in Seoul and Kyungkido, Korea were more interested in weight control than urban students [24]. Based on a previous study in West Malaysia, a higher number of rural adolescents (86.6%) were relatively knowledgeable regarding the importance of consuming vegetables as a source of fiber than urban adolescents (77.7%) [25]. These inconsistent findings regarding nutritional knowledge among urban and rural students might be influenced by socioeconomic differences [25,26] or by structural barriers such as limited health information access [27].

A small but significant difference in nutrition knowledge by gender was found and this supports previous findings. For example, a study among 348 secondary students aged 14–16 in Gujarat, demonstrated that females were more aware of food micronutrient components than males [28]. Moreover, female students’ knowledge regarding healthy eating, main food groups and vitamins or minerals tended to be higher than their male counterparts among 4700 primary school and junior high school pupils in Isfahan province, Iran [29]. Not surprisingly, female students were also reported to have better dietary habits such as consuming regular breakfast among college students in Ulsan, Korea [30]. In contrast, a study among 358 university students in Sarawak Malaysia, indicated no significant association between gender and knowledge except for items relating to protein and muscle building reflecting the potentially gendered interest in body building [31].

According to WHO guidelines, the HAZ and BAZ of students in Sabah are within normal limits. It is not surprising that the measured height for age Z score (HAZ), BMI, BMIz scores were significantly different between urban and rural students in this study. A similar finding was reported Liu et al. (2015) that children from urban *Hukou* (a household registration system that determines eligibility for various welfare benefits such as health care, education, housing, and employment in China) had a higher HAZ than children from rural hukou [32]. In another example, Herrador et al. (2014) reported that HAZ were below the WHO references value as well as a significant difference in the mean between Ethiopian urban and rural students [33]. HAZ measurement is an important indicator of nutritional status with an impact on scholastic performance. For example, HAZ measurements correlate with mathematics scores and cognitive functioning among students in Southeast Ethiopia [34]. Thus, our findings contribute to the existing literature that poorer nutritional status has the potential to impact both nutrition-related health and scholastic outcomes.

Our study indicated that a significantly higher number of urban students reported more interest in healthy and balanced foods, but this may simply reflect opportunity and accessibility of these foods compared to rural settings. A health seminar conducted among African-American high school students in Baltimore, Maryland USA, indicated health behavior changes including growing self-awareness and the changing roles as well as engagement with the community among the students [35]. It is proposed that a similar program could be conducted in Malaysia, especially among rural populations who may have less resources for healthy eating. Interestingly, the study indicated that fewer rural students reported the need to have a snack in between meals or eat outside the three main meals which warrants further investigation by conducting a focus group discussion. The study also showed that male students tend to choose a healthier and balanced diet compared with female students. This finding is rather surprising as females have been reported to be more interested in diet, nutrition and body weight among college and university students in other populations [36,37]. However, it should be noted that the difference between the level of awareness and attitude in gender might be attributed to social roles expectancy [38] and warranted further studies to obtain better insight.

A higher number of urban students ate breakfast daily and drank milk at least once a week compared to rural students. The finding in this study is similar to a previous study conducted among school students in Perak and Selangor states of West Malaysia, which indicated that a higher number of rural students consumed vegetables in their daily meal as compared with urban students [39]. Interestingly, Downs et al. (2012) [40] demonstrated that urban adolescents in America have higher vegetable consumption compared with rural adolescents due to the lower quality of fresh food available in rural areas. Another study in West Java, Indonesia demonstrated that lower income families appeared to have lower consumption of fruit and vegetable because of the additional cost of fruit and vegetables [41]. This is due to the fact that the marketing of vegetable commodities in certain parts of Indonesia consisted of multilevel network of marketing from farmers, collectors, groceries, market scalpers and wholesalers, before reaching the retailers and then the consumers, although most have worked as major vegetable producers. Similarly, few farmers in Sabah sell their vegetables directly to consumers. Similarly, urban school children in Bogor, Indonesia also showed better dietary habits indicated by greater number of school children with regular consumption of breakfast and dairy [42]. The differences in dietary patterns between urban and rural school students may be due to the fact that urban children had a higher chance to have regular breakfast, with parents of rural children not having the attitude that breakfast is important for children.

Interestingly, a higher consumption of vegetables and drinking milk was seen among female students. Other studies indicated parallel findings whereby female students demonstrated higher vegetable intake in their daily diet in Spain [43] and Indonesia [44]. Similarly, a cross-sectional study conducted among 4700 primary school and junior high school students in Isfahan province, Iran [29], showed that males were associated with lower vegetables and fruit consumption and higher intake of carbohydrates, fats and meats. It is widely accepted that vegetables and fruits consumption is associated with improved health outcomes, including cognitive and mental health [45]; thus, a better emphasis should be given to balancing the food consumption, particularly in males. In quest of emphasizing on healthy eating, various programs have been implemented in Malaysia, including “Eat Right, Be Positive about Your Body and Live Actively” (EPaL), specially aiming at preventing overweight and eating disorders among Malaysian adolescents [46]. Another programme is the School Nutrition Programme (SNP) which attempts to give nutritional education and build healthy school food environment [8]. Additionally, the Healthy School Canteen Guideline was developed and implemented by MOE since 2011 [47] to help canteen managers make healthier food and drink choices for school students.

Based on the classification of the strength of correlation between variables by Cohen 2013 [48], the present study showed a mild correlation between knowledge and practice among students which was expected because the knowledge scores consequently impact the nutritional practices. These positive correlations indicated that school students with better knowledge tend consume a better diet, for example as observed in Portugal [49]. The study also established no correlation between knowledge and attitude of urban and rural students, as supported by other related studies conducted in Tehran, Iran [50], South Sulawesi, Indonesia [44] and Noakhali district, Bangladesh [51]. Nonetheless, the phenomenon indicates that nutritional education should be further promoted to improve students’ behavior towards the importance of healthy nutrition.

Limitations of the study include that the districts and schools were selected purposively owing to the fact that Sabah is a large state, and some districts or schools are not accessible through land transportations which may limit the generalizability of the findings to all secondary school students in Sabah. Secondly, as reported elsewhere, reliance on self-report tends to bias towards more desired behaviors [52]. Nevertheless, efforts were made to minimize this limitation by ensuring that participants knew their responses were anonymous and unrelated to schoolwork. Finally, the handling of missing data of each individual may have the potential to create some bias.

## 5. Conclusions

Nutritional knowledge was similar among students in urban and rural settings. However, students in urban schools were more likely to eat breakfast each day and to report that healthy eating was a priority compared to rural students. Clearly there is a mismatch between knowledge and opportunity to practice this knowledge in more rural settings. Much work is needed to close the gap between nutrition knowledge, attitudes and practices and how this translates to healthy eating across settings and between genders. More effort into promoting nutrition education as well as translating this into action is needed. In future, engagement with parents is essential to translate school-based nutritional education to changes in diet at home. Educational workshops and seminars on nutrition are critical, to enhance nutrition knowledge so that even in settings with limited resources a healthy diet might be achieved.

## Figures and Tables

**Table 1:** Sociodemographic characteristics of secondary school students (N = 994).

Sociodemographic Variables	Total	Urban School (N = 792)	Rural School (N = 202)	*p*-Value
	(N, %)	(n, %)	(n, %)	
Age (years)				0.185
15	3 (0.003)	3 (0.38)	0	0.21
16	549 (55.23)	433 (54.67)	116 (57.43)	
17	427 (42.96)	341 (43.06)	86 (42.57)	
19	15 (1.51)	15 (1.89)	0	
Mean age (yr ± SD)	16.5 (0.59)	16.48 ± 0.61)	16.43 ± 0.5	
Form				NA
4	542 (54.53)	426 (53.79)	116 (57.43)	
5	437 (43.96)	351 (44.32)	86 (45.57)	
6	15 (1.51)	15 (1.89)	0 (0.00)	
Gender				0.17
Female	593 (59.66)	464 (58.59)	129 (63.86)	
Male	401 (40.34)	328 (41.41)	73 (36.14)	
Ethnicity				<0.001
*Kadazan-Dusun*	230 (23.16)	119 (15.04)	111 (54.95)	
*Bajau*	166 (16.72)	155 (19.60)	11 (5.45)	
Chinese	125 (12.59)	122 (15.42)	3 (1.49)	
Sabah Malay	125 (12.59)	123 (15.55)	2 (0.99)	
Brunei	94 (9.47)	90 (11.38)	4 (1.98)	
*Bugis*	35 (3.52)	34 (4.30)	1 (0.50)	
*Rungus*	25 (2.52)	7 (0.88)	18 (8.91)	
*Sungoi*	34 (3.42)	6 (0.76)	28 (13.86)	
^Others	159 (16.01)	135 (17.07)	24 (11.88)	
Height (cm) (mean ± SD)		159.4 (7.96)	156.2(13.2)	<0.0001
Height for age z score (HAZ) (mean ± SD)		−1.11 ± 0.82	−1.51 ± 1.76	<0.001
Height for age z score (HAZ) (median)		−1.13	−1.48	<0.001
Boys’ HAZ (mean ± SD)		−1.04 ± 0.80	−1.31 ± 0.67	0.01
Girls’ HAZ (mean ± SD)		−1.17 ± 0.84	−1.64 ± 2.14	<0.001
Weight (kg) (mean ± SD)		58.3 ± 20.3	53.9 ± 13.50	0.4
BMI (mean ± SD)		22.6 ± 5.00	21.76 ± 4.59	0.027
BMI for age Z score (BMIz) (mean ± SD)		1.08 ± 0.20	1.12 ± 0.19	0.019
BMIz (median)		1.1	1.15	0.008
Boys’ BMIz (mean ± SD)		1.05 ± 0.24	1.08 ± 0.23	0.352
Girls’ BMIz (mean ± SD)		1.1 ± 0.17	1.14 ± 0.15	0.024

Note: cm = centimeter; SD = standard deviation; BMI = body mass index; BMI for age Z score = BMIz. ^Others: other ethnicities include *Murut*, *Tidong*, *Cocos*, *Lundayeh*, *Iranun* and *Suluk*. A number of the participants are also of mixed parentage between Chinese (*Sino-peranakan*), Indian and Eurasian.

**Table 2:** Student nutritional knowledge based on locality of schools and gender.

Variables	n	Mean ± SD	Median	25th Percentile	75th Percentile	Minimum	Maximum	*p*-Value
Type of school								
Urban	792	18.59 ± 3.14 *^,†^	19	17	21	1	24	
Rural	202	18.59 ± 2.66 *^,†^	19	17	20	11	23	0.986
Gender								
Male	401	18.1 ± 3.4 *^,†^	16	19	21	1	24	
Female	593	18.9 ± 2.8 *^,†^	18	19	21	7	24	<0.0001

Note: SD, standard deviation; * Kruskal–Wallis test (*p* < 0.0001); ^†^, Bonferroni’s post hoc test.

**Table 3:** Nutritional attitudes and their association with locality of schools and gender and students.

Attitudes	Variable	n, % of Response or Mean ± SD					*p*-Value
		Strongly Disagree	Disagree	Not Sure	Agree	Strongly Agree	
**General Attitude**							
Healthy and balanced foods are my priorities when choosing foods	Urban Rural	4 (0.51) 1 (0.50)	42 (5.34) 19 (9.50)	272 (34.61) 83 (41.50)	326 (41.48) 63 (31.50)	142 (18.07) 34 (17.00)	0.030
	Female Male	3 (0.51) 2 (0.51)	41 (6.94) 20 (5.06)	232 (39.26) 123 (31.14)	219 (37.06) 170 (43.04)	96 (16.24) 80 (20.25)	0.041
I still follow the main meals (breakfast, lunch and dinner) even though I am busy	Urban Rural	23 (2.93) 4 (1.98)	158 (20.10) 52 (25.74)	297 (37.79) 85 (42.08)	200 (25.45) 41 (20.30)	108 (13.74) 20 (9.90)	0.122
	Female Male	17 (2.87) 10 (2.53)	133 (22.43) 77 (19.49)	244 (41.15) 138 (34.94)	131 (22.09) 110 (27.85)	68 (11.47) 60 (15.19)	0.057
**Appetite**							
Appetite is my own priority when choosing foods	Urban Rural	15 (1.91) 3 (1.49)	73 (9.29) 20 (9.90)	112 (14.25) 22 (10.89)	383 (48.73) 108 (53.47)	203 (25.83) 49 (24.26)	0.655
	Female Male	9 (1.52) 9 (2.28)	47 (7.93) 46 (11.65)	73 (12.31) 61 (15.44)	310 (52.28) 181 (45.82)	154 (25.97) 98 (24.81)	0.089
Between main meals, I need a snack to prevent from being hungry	Urban Rural	68 (8.66) 6 (2.97)	235 (29.94) 56 (27.72)	220 (28.03) 54 (26.73)	189 (24.08) 69 (34.16)	73 (9.30) 17 (8.42)	0.008
	Female Male	44 (7.42) 30 (7.61)	171 (28.84) 120 (30.46)	168 (28.33) 106 (26.90)	153 (25.80) 105 (26.65)	57 (9.61) 33 (8.38)	0.930
Sweet foods are my favorite food	Urban Rural	43 (5.51) 10 (4.95)	250 (32.05) 64 (31.68)	228 (29.23) 44 (21.78)	200 (25.64) 63 (31.19)	59 (7.56) 21 (10.40)	0.150
	Female Male	34 (5.76) 19 (4.85)	187 (31.69) 127 (32.40)	158 (26.78) 114 (29.08)	168 (28.47) 95 (24.23)	43 (7.29) 37 (9.44)	0.438
Regardless of the time, I eat anytime I want	Urban Rural	137 (17.50) 45 (22.28)	265 (33.84) 75 (37.13)	215 (27.46) 35 (17.33)	119 (15.20) 32 (15.84)	47 (6.00) 15 (7.43)	0.049
	Female Male	119 (20.10) 63 (16.03)	205 (34.63) 135 (34.35)	145 (24.49) 105 (26.72)	94 (15.88) 57 (14.50)	29 (4.90) 33 (8.40)	0.113

Note: SD, standard deviation.

**Table 4:** Nutritional practices and their association with locality of schools and gender.

Practice	Variable	n, % of Response					*p*-Value
		Everyday	2–3 Times Per Week	Once in a Week	2–3 Times Per Month	Never	
How frequently do you eat your breakfast?	Type of school						
	Urban	567 (72.14)	158 (20.10)	33 (4.20)	14 (1.78)	14 (1.78)	< 0.001
	Rural	101 (50.25)	70 (34.83)	19 (9.45)	8 (3.98)	3 (1.49)	
	Gender						
	Male	278 (70.56)	86 (21.83)	13(3.30)	8 (2.03)	9 (2.28)	0.116
	Female	390 (65.77)	142 (23.95)	39 (6.58)	14 (2.36)	8 (1.35)	
How frequently do you eat snacks?	Type of school						
	Urban	262 (33.38)	368 (46.88)	81 (10.32)	67 (8.54)	7 (0.89)	0.166
	Rural	51 (25.50)	109 (54.50)	25 (12.50)	14 (7.00)	1 (0.50)	
	Gender						
	Male	110 (27.99)	199 (50.64)	47 (11.96)	33 (8.40)	4 (1.02)	0.303
	Female	203 (34.29)	278 (46.96)	59 (9.97)	48 (8.11)	4 (0.68)	
Indicate the level of your favorite fast foods such as burgers, sausage, pizza and fried chicken?	Type of school						
	Urban	8 (1.02)	16 (2.04)	90 (11.45)	413 (52.54)	259 (32.95)	0.451
	Rural	0 (0.00)	6 (3.05)	19 (9.64)	101 (51.27)	71 (36.044)	
	Gender						
	Male	4 (1.02)	7 (0.70)	40 (10.15)	219 (55.58)	124 (31.47)	0.448
	Female	4 (0.68)	15 (2.55)	69 (11.71)	295 (50.08)	206 (34.97)	
How often do you eat fast foods such as burgers, sausage, pizza and fried chicken?	Type of school						
	Urban	44 (5.62)	314 (40.10)	169 (21.58)	247 (31.55)	9 (1.15)	0.318
	Rural	7 (3.52)	95 (47.74)	36 (18.09)	59 (29.65)	2 (1.01)	
	Gender						
	Male	31 (7.93)	163 (41.69)	84 (21.48)	106 (27.11)	7 (1.79)	0.004
	Female	20 (3.38)	246 (41.62)	121 (20.47)	200 (33.84)	4 (0.68)	
How often do you eat vegetables?	Type of school						
	Urban	463 (58.91)	209 (26.59)	37 (4.71)	39 (4.96)	38 (4.83)	
	Rural	132 (66.67)	51 (25.76)	9 (4.55)	5 (2.53)	1 (0.51)	0.024
	Gender						
	Male	217 (55.08)	118 (29.95)	18 (4.57)	26 (6.60)	15 (3.81)	0.011
	Female	378 (64.07)	142 (24.07)	28 (4.75)	18 (3.05)	24 (4.07)	
How often do you drink milk?	Type of school						
	Urban	165 (21.05)	290 (36.00)	85 (10.84)	177 (22.58)	67 (8.55)	0.002
	Rural	19 (9.50)	83 (41.50)	30 (15.00)	44 (22.00)	24 (12.00)	
	Gender						
	Male	93 (23.66)	150 (38.17)	41 (10.43)	83 (21.12)	26 (6.62)	0.005
	Female	91 (23.66)	223 (37.73)	74 (12.52)	138 (23.35)	65 (11.00)	

**Table 5:** Correlation of KAP and locality of school.

KAP Domain	Total		Urban		Rural		Male		Female	
	r	*p*-Value	r	*p*-Value	r	*p*-Value	r	*p*-Value	r	*p*-Value
Knowledge-attitude	−0.05	0.0980	−0.05	0.1486	−0.08	0.2807	−0.07	0.1839	−0.03	0.4631
Knowledge-practice	−0.125	0.0001	−0.14	0.0001	−0.03	0.6638	−0.12	0.0209	−0.14	0.0007
Attitude-practice	−0.13	<0.0001	−0.13	0.0004	−0.13	0.0794	−0.14	0.0075	−0.12	0.0039

Note: r, correlation coefficient.

## Data Availability

Data is contained within the article.
